# Targeted alpha therapy using astatine (^211^At)-labeled phenylalanine: A preclinical study in glioma bearing mice

**DOI:** 10.18632/oncotarget.27552

**Published:** 2020-04-14

**Authors:** Tadashi Watabe, Kazuko Kaneda-Nakashima, Yoshifumi Shirakami, Yuwei Liu, Kazuhiro Ooe, Takahiro Teramoto, Atsushi Toyoshima, Eku Shimosegawa, Takashi Nakano, Yoshikatsu Kanai, Atsushi Shinohara, Jun Hatazawa

**Affiliations:** ^1^Department of Nuclear Medicine and Tracer Kinetics, Graduate School of Medicine, Osaka University, Suita, Japan; ^2^Institute for Radiation Sciences, Osaka University, Suita, Japan; ^3^Core for Medicine and Science Collaborative Research and Education, Project Research Center for Fundamental Sciences, Graduate School of Science, Osaka University, Toyonaka, Japan; ^4^Department of Molecular Imaging in Medicine, Graduate School of Medicine, Osaka University, Suita, Japan; ^5^Research Center for Nuclear Physics, Osaka University, Ibaraki, Japan; ^6^Department of Bio-system Pharmacology, Graduate School of Medicine, Osaka University, Suita, Japan; ^7^Department of Chemistry, Graduate School of Science, Osaka University, Toyonaka, Japan

**Keywords:** alpha therapy, astatine, glioma, LAT1, phenylalanine

## Abstract

Phenylalanine derivatives, which target tumors especially through L-type amino acid transporter-1 (LAT1), have elicited considerable attention. In this study, we evaluated the treatment effect of phenylalanine labeled with the alpha emitter astatine (^211^At-PA) in tumor bearing mice. The C6 glioma, U-87MG, and GL261 cell lines were subjected to a cellular ^211^At-PA uptake analysis that included an evaluation of the uptake inhibition by the system L amino acid transporter inhibitor 2-aminobicyclo-(2,2,1)-heptane-2-carboxylic acid (BCH). BCH significantly inhibited para-^211^At-PA uptake in C6 glioma (12.2 ± 0.8%), U-87MG (27.6 ± 1.1%), and GL261 (12.6 ± 2.0%) cells compared to baseline, suggesting an uptake contribution by system L amino acid transporters. Subsequently, xenograft and allograft models were prepared by subcutaneously injecting C6 glioma (*n* = 12) or GL-261 cells (*n* = 12), respectively. C6 glioma mice received three ^211^At-PA doses (0.1, 0.5, or 1 MBq, *n* = 3/dose), while GL261 mice received one high dose (1 MBq, *n* = 7). ^211^At-PA exhibited a tumor growth suppression effect in C6 glioma models in a dose-dependent manner as well as in GL-261 models. This phenylalanine derivative labeled with astatine may be applicable as an alpha therapy that specifically targets system L amino acid transporters.

## INTRODUCTION

Targeted alpha therapy has received attention as an effective form of radionuclide treatment, particularly for refractory and/or recurrent malignant tumors such as malignant glioma. Glioma, a form of brain malignancy, is highly refractory and associated with a high relapse rate and poor prognosis [1]. Currently, the standard treatment for glioma is surgery, followed by chemotherapy and radiation therapy according to the histological type or aggressiveness of the tumor [1, 2]. In a previous report, however, patients with glioblastoma, a type of glioma, had a 5-year overall survival rate of only 9.8% after temozolomide therapy [2]. These observations emphasize the need for more effective treatments, including targeted alpha therapy, which would improve the prognosis of affected patients.

Many malignant tumors, including glioma, exhibit upregulated amino acid transport and glucose transport [3]. Accordingly, amino acid probes for diagnostic positron emission tomography (PET), such as ^11^C-methionine (^11^C-MET), ^18^F-fluorodihydroxyphenylalanine (^18^F-DOPA), and ^18^F-fluoroethyltyrosine (^18^F-FET), are used mainly to evaluate the extent of tumor invasion and differentiate recurrence from radiation necrosis [3, 4]. Additionally, phenylalanine (PA) derivatives such as ^18^F-fluoro-borono-PA (^18^F-FBPA) and 4-borono-L-PA (BPA) specifically target tumors and are used mainly in the context of boron neutron capture therapy [5]. They were both reported to be substrates of the L-type amino acid transporter 1 (LAT1) and were found to be taken up selectively by tumors, with minimal physiological accumulation in normal organs [5, 6]. The derivative labelled with the gamma emitting isotope, para-^123^I-iodo-L-phenylalanine (^123^I-IPA), has been used with some success as a diagnostic imaging agent in glioblastoma patients [7]. The corresponding ^131^I derivative (^131^I-IPA) has been studied as a therapeutic agent, and is currently being advanced as a combination therapy with external beam radiation in a PhaseI/II clinical trial [8, 9]. These PA-based compounds exhibit characteristics of high tumor to normal brain ratio that would be ideal for a targeted alpha therapeutic strategy.

Longer half-life alpha emitters, such as actinium (^225^Ac, half-life: 10 day) could potentially induce long-term renal toxicity due to recoiled daughter radionuclides, such as ^213^Bi [10]. In contrast, the alpha emitter astatine (^211^At) is a halogen element with a relatively short half-life (7.2 hr) and simple decay chain. ^211^At can be produced in a commercially available medium-energy cyclotron by alpha beam irradiation of a bismuth target [11]. In addition, ^211^At can be combined with small molecule compounds to enable rapid distribution to the target. In this study, we evaluated the selectivity of ^211^At-para-astato-L-PA (^211^At-PA) for amino acid transporters focusing on LAT1, as well as the treatment effect of this derivative in mouse glioma xenograft and allograft models.

## RESULTS

**Figure 1:**
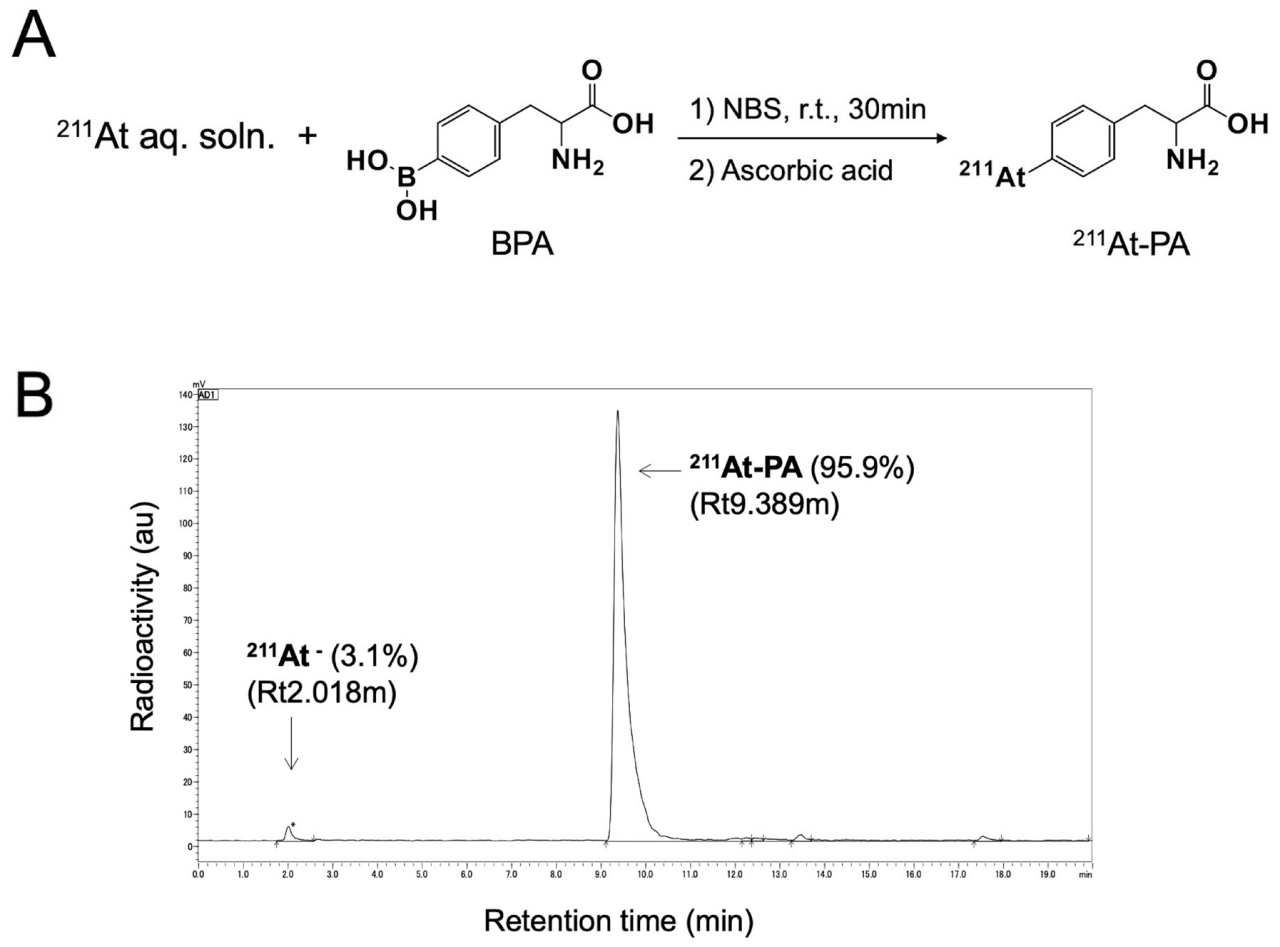
(**A**) Reaction scheme for the labeling of para-borono-L-phenylalanine (BPA) with ^211^At (represented by para-^211^At-PA). (**B**) Radiochromatogram obtained by eluting a solid-phase extraction-purified para-^211^At-PA solution on a reverse-phase high-performance liquid chromatography column under a gradient.

A nucleophilic substitution reaction between the boronic groups and astatine was used to radiolabel para- and meta-BPA with ^211^At, yielding para- and meta-^211^At-PA, respectively (Figure 1A). The corresponding radiochemical yields and the radiochemical purities were > 80% and > 94%, respectively. Figure 1B depicts a radiochromatogram of para-^211^At-Phe. Both products remained stable in vials for 24 hr. No other side products were observed.

**Figure 2:**
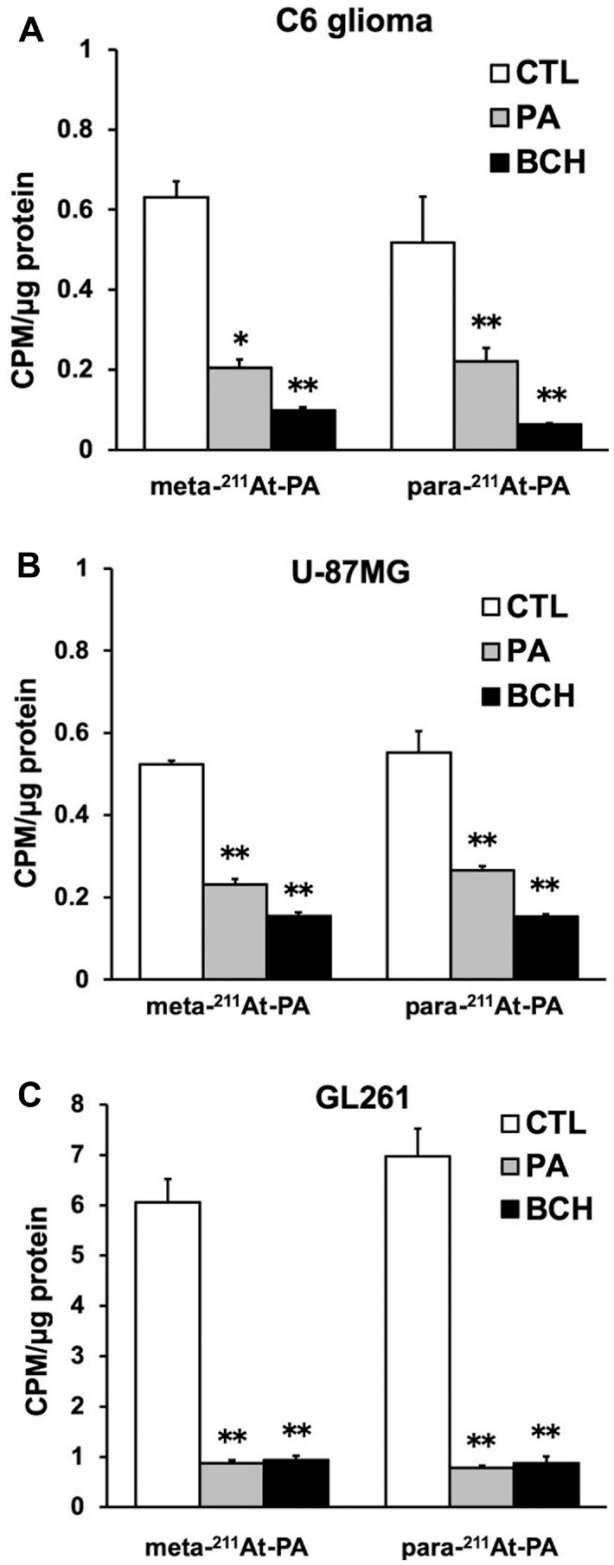
Meta- and para- ^211^At-PA transport in an *in vitro* cellular uptake and inhibition assay conducted in the absence or presence of 1 mM non-radiolabeled phenylalanine (PA) or 20 mM 2-aminobicyclo-(2,2,1)-heptane-2-carboxylic acid (BCH; system L amino acid transporter inhibitor). (**A**) C6 glioma, (**B**) U-87MG, and (**C**) GL261 cell lines [^*^ *p* < 0.05 and ^**^ *p* < 0.01 compared with control (CTL)]. Uptake data are expressed as mean values ± standard errors of the means (four replicates per condition).

**Figure 3:**
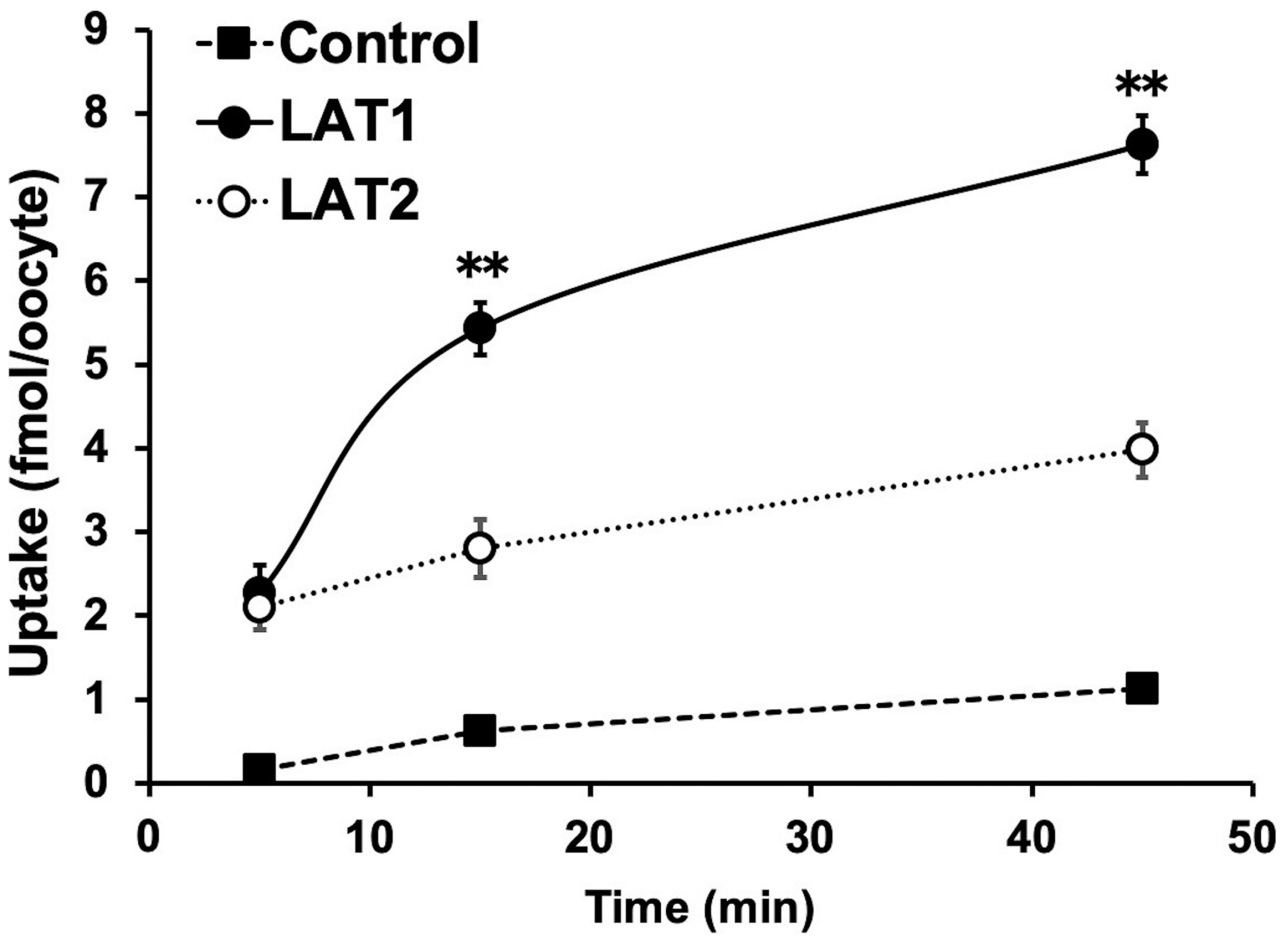
Analysis of para-^211^At-phenylalanine (PA) transport by L-type amino acid transporter-1 (LAT1) and LAT2. Para-^211^At-PA uptake by control oocytes (black square) and oocytes expressing LAT1 (black circle) or LAT2 (white circle) in choline buffer was measured for 5, 15, and 45 min. The uptake rates are expressed as mean values ± standard errors of the means (*n* = 9–11; ^**^ *p* < 0.01 in a comparison between LAT1 and LAT2).

In the cellular uptake analysis, the addition of BCH significantly inhibited uptake by C6 glioma (12.2 ± 0.8%), U-87MG (27.6 ± 1.1%), and GL261 cells (12.6 ± 2.0%), suggesting that ^211^At-PA uptake is predominantly mediated by system L amino acid transporters (Figure 2). As no major differences were observed in a comparison between meta-^211^At-PA and para-^211^At-PA, we used the latter in evaluations of treatment effects. Figure 3 demonstrates the transport of para-^211^At-PA into oocytes by LAT1 and LAT2. Significantly greater transport of para-^211^At-PA was observed with LAT1 than with LAT2, indicating a larger contribution of the former to the uptake of this PA derivative.

**Figure 4:**
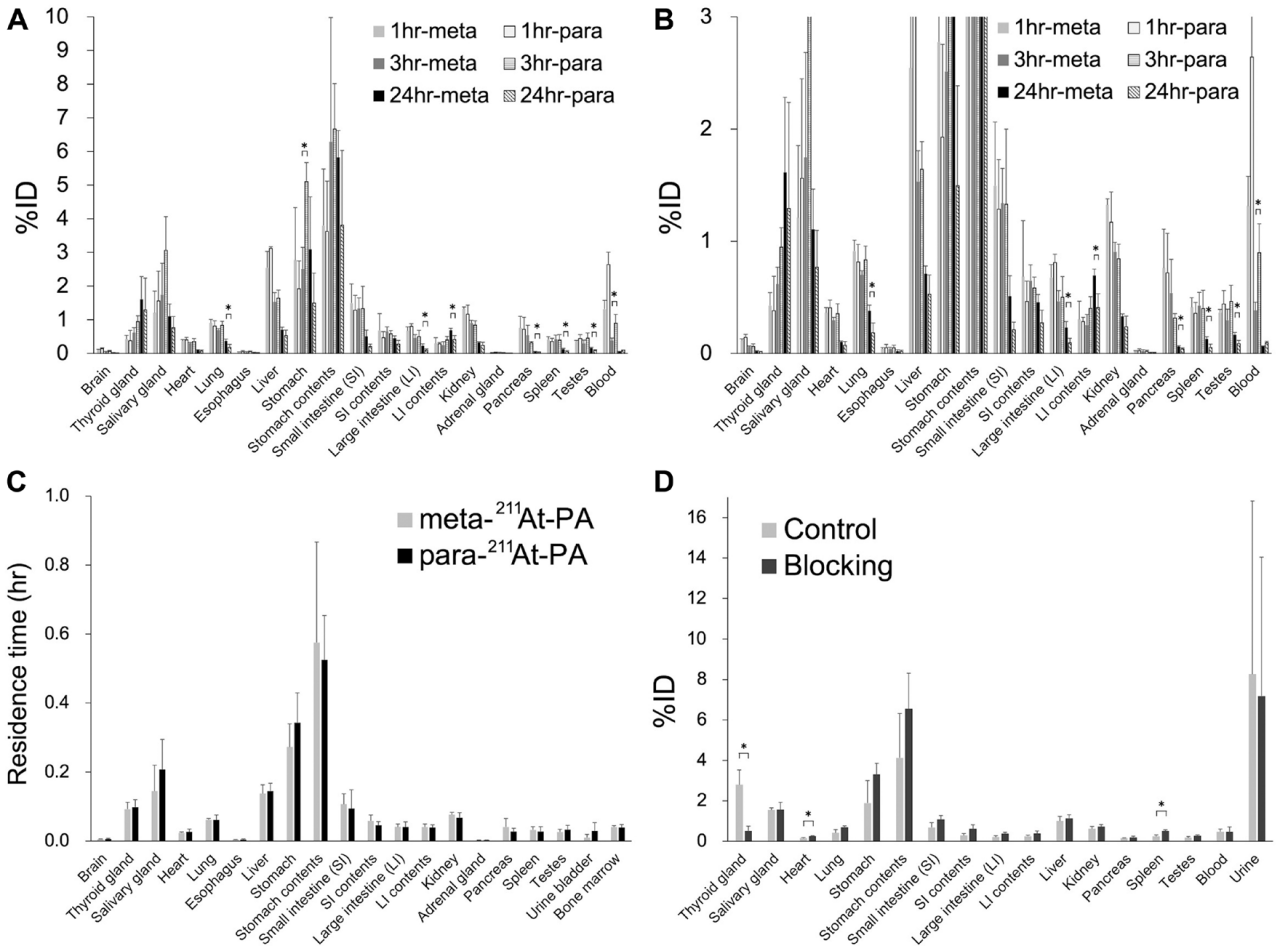
Whole-body distribution of meta-^211^At-phenylalanine (PA) and para-^211^At-PA in normal ICR mice after intravenous administration. (**A**, **B**) Percent injected doses (%; note the difference in vertical scales between A and B) (*n* = 3 for each time point of each group) and (**C**) residence time (hr) in major organs. (**D**) Effect of iodine blocking after the intravenous administration of para-^211^At-PA (*n* = 3 for each group). Data are expressed as mean values with standard deviations (^*^ *p* < 0.05).

The whole-body distributions of meta-^211^At-PA and para-^211^At-PA were evaluated in normal ICR (Figure 4A and 4B). Although no significant difference in the distributions of meta-^211^At-PA and para-^211^At-PA was observed at 1 hr post-injection, significant differences were observed in the stomach and blood at 3 hr and in the lung, large intestine (LI), contents of the LI, pancreas, spleen, and testes at 24 hr. However, residence time showed comparable results between meta-^211^At-PA and para-^211^At-PA in all the major organs examined in this study (Figure 4C), reflecting the similar early distribution. Iodine blocking led to a significant decrease (81%) in the accumulation of para-^211^At-PA in the thyroid and increased accumulation in the heart and spleen (Figure 4D).

**Figure 5:**
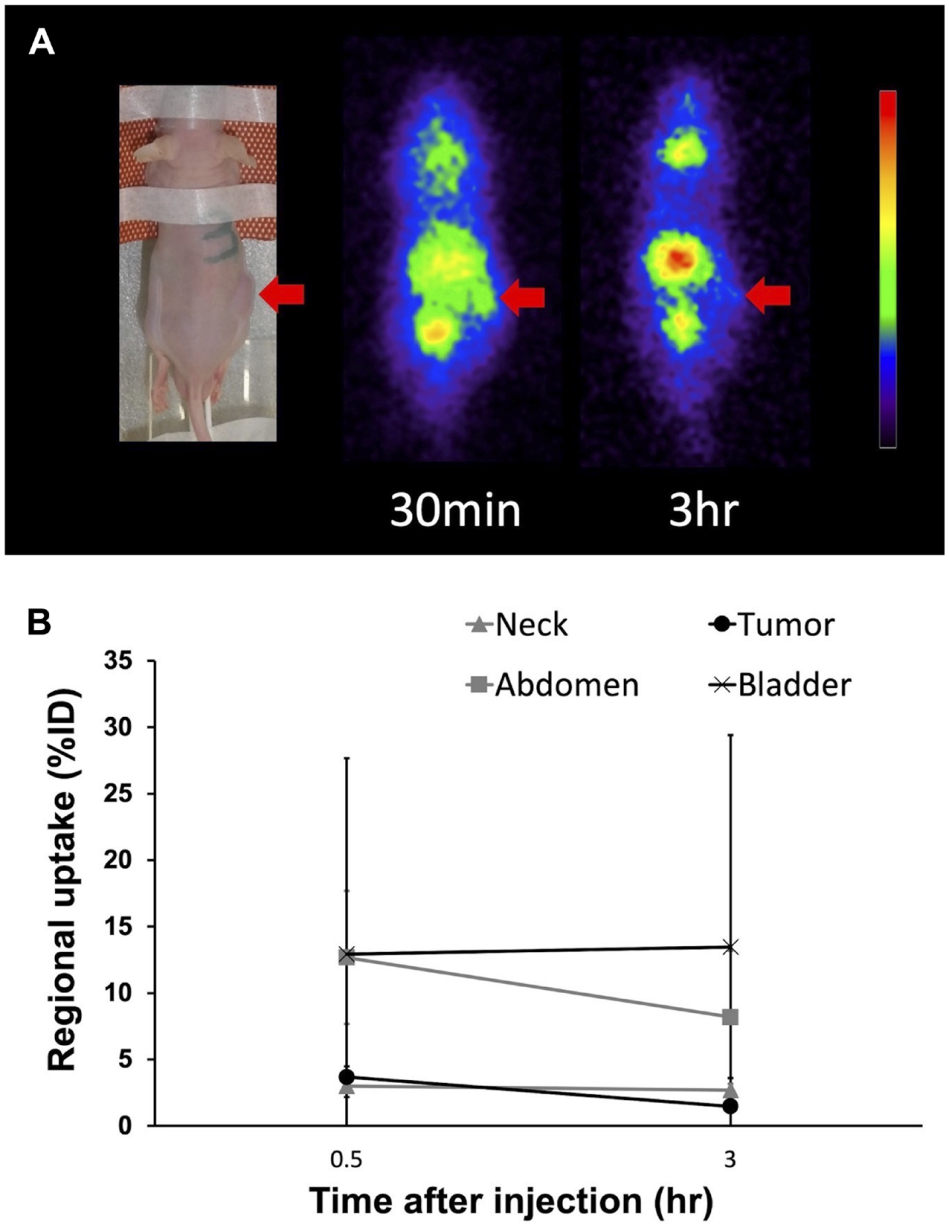
(**A**) Planar images of a C6 glioma xenograft model mouse at 30 min and 3 hr after the injection of para-^211^At-phenylalanine (PA). Arrows indicate the C6 glioma xenograft. (**B**) Uptake in the tumor and other regions as determined from an analysis of regions of interest on planar images.

**Figure 6:**
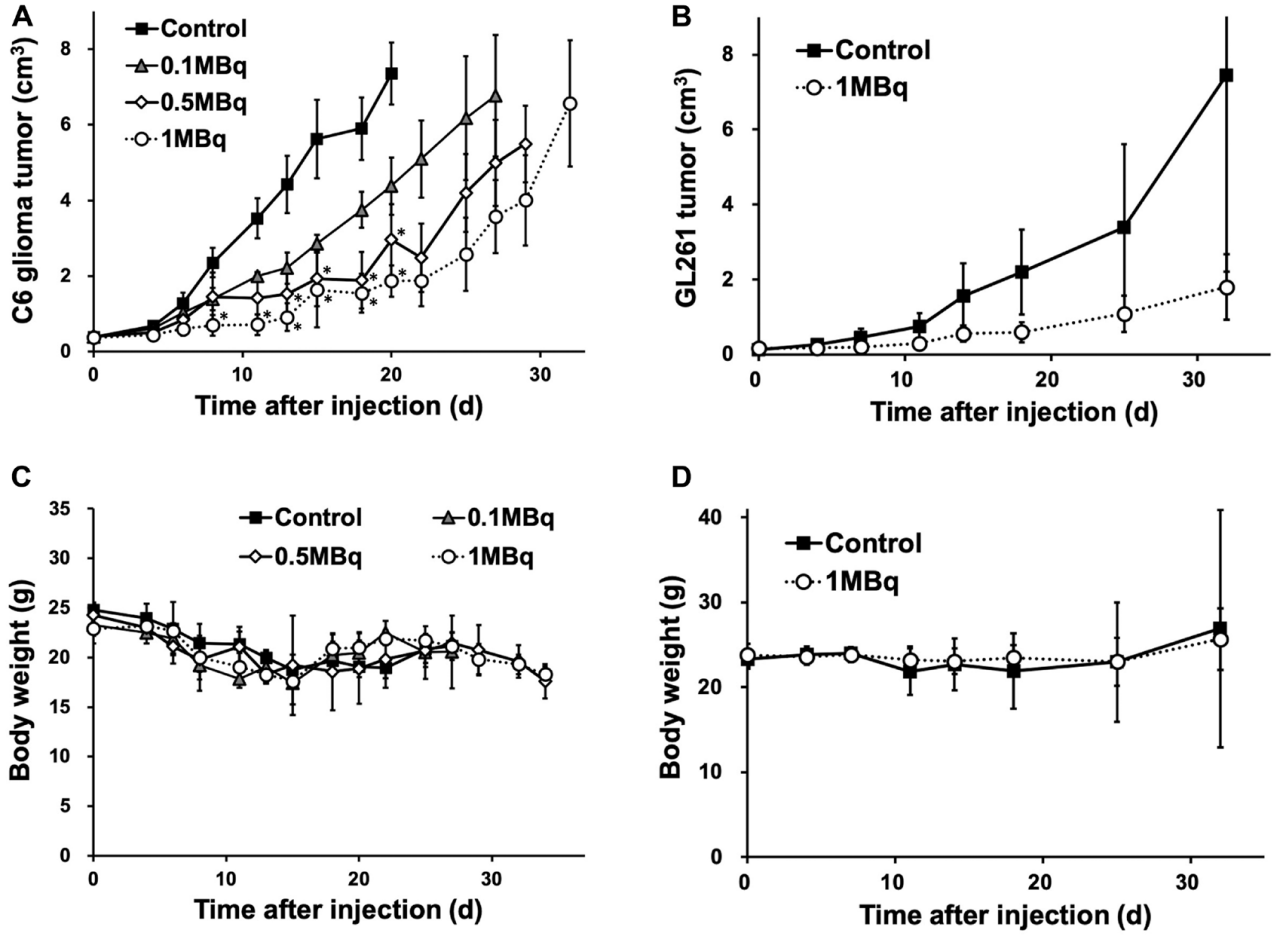
(**A**, **B**) Tumor growth suppression effect represented by tumor size and (**C**, **D**) changes in body weight in C6 glioma and GL261 allograft mice after the injection of para-^211^At-phenylalanine (PA) (^*^ *p* < 0.05 compared to the control).

Planar imaging of C6 glioma-bearing mice revealed the early distribution and gradual washout of para-^211^At-PA in the tumors (3.7 ± 0.8%ID at 30 min and 1.5 ± 0.2%ID at 3 hr), with an estimated absorbed dose of 1.7 ± 0.6 Gy/MBq (Figure 5). We could not detect the tumor uptake by planar imaging in the GL261 tumor model, since the GL261 allograft tumors were smaller (0.15 ± 0.06 cm^3^) compared to the C6 glioma xenograft tumors (0.39 ± 0.05 cm^3^). In an evaluation of the treatment effect, para-^211^At-PA suppressed the growth of C6 glioma xenograft tumors in a dose-dependent manner, with tumor size ratios (relative to the control) of 0.60, 0.40, and 0.25 at 3 weeks post-injection with 0.1, 0.5, or 1 MBq of para-^211^At-PA, respectively (Figure 6A). Similar tumor growth suppression was also observed in GL261 allograft mice (relative tumor size: 0.24 at day 32) (Figure 6B). No significant differences were observed with respect to the changes in body weights between para-^211^At-PA-injected and control mice in either the C6 glioma xenograft or GL261 allograft model (Figure 6C, 6D).

## DISCUSSION

We have demonstrated the potential efficacy of ^211^At-PA as a targeted alpha therapeutic agent for glioma. We further confirmed the transport of this PA derivative into cells via system L amino acid transporters, particularly LAT1. *In vivo*, we observed para-^211^At-PA dose-dependent tumor growth suppression in a mouse xenograft model of C6 glioma and relatively slower tumor regrowth in a GL261 tumor-bearing mice. In addition, we used a borono-precursor to achieve the ^211^At labeling of PA. This preparation method proceeded efficiently under mild conditions in aqueous media and did not require any toxic reagents, in contrast to previous work [12–14].

In our previous studies, we demonstrated high levels of accumulation of various amino acid PET probes, such as ^18^F-FBPA, ^18^F-FAMT and ^11^C-MET, in C6 glioma. Moreover, we demonstrated the functional expression of LAT1 in these tumors using histology and western blot analyses [5, 15]. A western blot analysis of GL261 cells also revealed strong expression of LAT1 [16]. In our current *in vitro* cellular uptake analysis, we demonstrated the transport of ^211^At-PA via system L amino acid transporters. In a previous study of a radioiodine-labelled PA with similar kinetics as ^211^At-PA, transport was mediated predominantly by the L and ASC amino-acid transport systems [12, 17]. Other studies of glioma, including recurrent cases, have also reported the high uptake of ^18^F-FBPA, ^18^F-FAMT, and ^11^C-MET [3, 18, 19]. Moreover, Youland and colleagues reported that LAT1 expression (evaluated via immunofluorescence) correlated with ^18^F-DOPA uptake in newly diagnosed human astrocytoma [20]. Taken together, these observations suggest that the use of alpha emitters to target system L amino acid transporters represents a promising treatment strategy for glioma patients. As LAT1 expression has been observed in many other types of cancer [21], ^211^At-PA could potentially be used in a universal cancer treatment strategy.

Borrmann et al. reported that intravenous treatment with ^211^At-PA improved the health conditions and enhanced the survival durations of rats with intracranial glioblastomas [13]. In this study, we studied subcutaneously implanted tumors to overcome the short observation period and challenges associated with tumor size monitoring in an intracranial implantation model. To further enable our study, we selected the C6 glioma and GL261 cell lines, which are well-established and have similar ^11^C-Met transport ability (data not shown).

In the cellular uptake analysis, GL261 cells exhibited approximately 10 times higher uptake than that in other cell lines. Since GL261 cells are usually cultured in a medium with a high amino acid concentration, it is presumed they have a high capacity for amino acid uptake compared to other cell lines. Although there was no significant difference in the *in vitro* cellular uptake and the residence time of meta-^211^At-PA and para-^211^At-PA in normal organs, BCH treatment showed a small trend towards greater inhibition (% inhibition compared to the control) of para-^211^At-PA uptake compared to meta-^211^At-PA in all cell-lines (Figure 2). Therefore, we thought that para-^211^At-PA uptake could be more specific to system L amino acid transporters. In addition, para-iodo-L-phenylalanine labelled with ^123^I/^131^I has been reported to show high uptake in glioma patients [7–9], suggesting the best coupling to be para-iodo/astato-L-phenylalanine.

One major challenge to the use of ^211^At-PA is the distribution of free ^211^At-astatide in the body. ^211^At-astatide is thought to be deastatinated during metabolic processes. We have previously reported the specific uptake of astatide in the thyroid gland through the sodium iodide symporter (NIS), in a manner similar to iodide, and identified the thyroid gland as an organ at risk [11]. We subsequently demonstrated that the thyroid uptake of ^211^At-astatide, which was deastatinated from ^211^At-PA, could be reduced by 80% following the pre-administration of iodine [22]. Furthermore, no significant changes in body weight were observed in the tumor bearing mice in this study. Although a more detailed investigation of side effects is needed, the effect of free ^211^At-astatide does not appear to be significant, especially in the context of refractory glioma with a poor prognosis.

We further observed the rapid uptake of para-^211^At-PA by the tumor, which reflects the advantage of a small molecule compound. However, a planar imaging analysis revealed a slow washout from the C6 glioma xenograft between 30 min and 3 hr post-injection. This washout is attributable to an efflux component in the transport of ^211^At-PA due to the bidirectional nature of the amino acid transporters reflecting the decreased concentration in the blood, and was consistent with our previous studies of amino acid PET probe kinetics in C6 glioma xenografts [4, 12]. Despite this washout, we observed an anti-tumor effect in the xenograft models. The estimated absorbed dose in the C6 glioma xenograft was 1.7 ± 0.6 Gy/MBq. In our previous study using ^211^At-NaAt, the estimated dose was 9.7 ± 7.0 Gy/MBq in the K1-NIS xenograft, which showed better tumor retention [11]. Regarding the treatment effect, ^211^At-NaAt treatment showed tumor regression followed by the delayed regrowth, whereas ^211^At-AtPA treatment showed tumor growth suppression in a dose dependent manner. Although the treatment effect of ^211^At-AtPA in glioma is smaller compared to that of ^211^At-NaAt in K1-NIS due to its faster clearance, we could deliver a certain dose in the glioma xenograft.

Therefore, these ^211^At-labelled compounds, which have short durations of action, should be considered effective treatment options if distribution to the target occurs rapidly during the period of high radioactivity before decay. The repeated administration of ^211^At-PA at appropriate intervals may also enable the effective treatment of glioma. These possible clinical applications should be evaluated in future studies, along with a detailed examination of the associated toxicities and an evaluation of long-term survival. Regarding the clinical translation and patient impact, ^211^At-PA will be beneficial to patients with refractory glioma, especially those with highly invasive and spreading tumors. It is expected that ^211^At-PA treatment will have a substantial impact on the management of glioma patients and improve the prognosis.

In conclusion, we have demonstrated the cellular uptake of ^211^At-PA and the tumor growth suppression effects of this PA derivative in mouse xenograft and allograft models of malignant glioma. Our findings suggest that ^211^At-PA could be useful as an alpha therapy specific for system L amino acid transporters expressed on malignant tumors.

## MATERIALS AND METHODS

### Production of ^211^At and synthesis of ^211^At-PA

^211^At was procured from the Research Center for Nuclear Physics at Osaka University and RIKEN via Supply Platform of Short-lived Radioisotopes [11]. ^211^At was produced in the ^209^Bi (α,2n)^211^At reaction using the AVF Cyclotron. The metallic Bi target was prepared by a vacuum evaporation method. The produced ^211^At was separated and purified via dry distillation from the bismuth target. The irradiated Bi target was put in a quartz column and heated up to 850°C using an electric tubular furnace under mixed helium and oxygen gas flow. The evaporated ^211^At was then swept out from the quartz column with the mixed gas flow and was passed through a Teflon tube which was cooled by ice water to trap evaporated ^211^At. After collection of ^211^At on the Teflon trap tube for approximately 20 of minutes, the trapped ^211^At was dissolved in approximately 100 μL of distilled water. The radioactivity of ^211^At dissolved in distilled water was determined by measurement of X-ray from a daughter nuclide, ^211^Po, with a high-purity germanium (HPGe) detector.

Ascorbic acid, para-borono-L-phenylalanine (para-BPA), meta-borono-D, L-phenylalanine (meta-BPA), *N*-bromosuccinimide (NBS), and an injectable solution of 7% sodium hydrogen carbonate (7% NaHCO_3_) were purchased from Nacalai Tesque (Kyoto, Japan). For the experiments, 1 mg of para-BPA (or meta-BPA) was dissolved in 0.1 mL of 7% NaHCO_3_. Next, 0.1 mL of the 1% (w/v) para-BPA (or meta-BPA) solution and an aliquot of ^211^At aqueous solution (10–20 MBq) were poured into a glass vial, stirred, and mixed with 30 μL of an aqueous solution of 0.4% (w/v) NBS for 30 min at room temperature. Finally, 30 μL of an aqueous solution of 3% (w/v) ascorbic acid was added to the mixture to stop the oxidation reaction, and the mixture was purified using solid-phase extraction. The purified para-^211^At-PA or meta-^211^At-PA was then analyzed using a high-performance liquid chromatography system (Shimazdu, Kyoto) equipped with a reverse-phase Cosmosil column (5C18 MS2, 150 mm × 4.6 mm; Nacalai Tesque). The products were eluted using a gradient solvent system (0–100% acetonitrile in water) for 20 min and then measured using a gamma-ray detector (GABI star, Elysia-raytest, Germany) and a UV detector at 254 nm.

The same method was used to synthesize para-iodo-L-phenylalanine separately, using equivalent amounts of BPA and sodium iodide. The chemical formula of this product was determined via a liquid chromatography-mass spectrometry analysis to be para-iodo-L-phenylalanine (extracted mass of I-Phe+1: 291.983 Da).

### *In vitro* cellular uptake and inhibition assay

The C6 rat glioma cell line was provided by RIKEN BRC (Tsukuba, Japan) and cultured in F-12K medium (Gibco, Grand Island, NY, USA) containing 10% fetal bovine serum (FBS; Sigma-Aldrich). The U-87 MG human glioblastoma cell line was purchased from American Type Culture Collection (Manassas, VA, USA) and cultured in Modified Eagle’s medium (MEM; Sigma-Aldrich, St. Louis, MO, USA) containing 1% Non Essential Amino Acids (NEAA) + 1 mM Sodium Pyruvate (NaP) and 10% FBS. The GL261 murine glioma cell line was provided by the *In Vivo* Cellular and Molecular Imaging Lab at Vrije Universiteit, Brussels and cultured in high-glucose Dulbecco’s MEM (DMEM; Wako Pure Chemical, Osaka, Japan) containing 10% FBS. All cells were maintained at 37°C in a humidified incubator containing 5% CO_2_ and were washed with phosphate-buffered saline (PBS) and harvested with trypsin as necessary. LAT1 expression of these cells were confirmed in previous studies [16, 23].

Initially, the cells were seeded onto a 24-well plate (density: 1 × 10^5^/well) and cultured for 2 days. The uptake of ^211^At-PA was inhibited by adding either 1 mM unlabeled PA as competitor or 20 mM 2-aminobicyclo-(2,2,1)-heptane-2-carboxylic acid (BCH), an inhibitor of system L amino acid transporters 5 min before adding ^211^At-PA. The amount of BCH was more than 100 times of ^211^At-PA, and the dose of ^211^At-PA was approximately 10 kBq/well. After incubation with para- or meta-^211^At-PA, the cells were washed twice with PBS for removing the component which did not internalize to the cells, and the retained radioactivity was measured using a 2480 Wizard^2^ gamma counter (Perkin Elmer, Waltham, MA, USA). Counts per minute (CPM) of the samples were measured by gamma counter. The protein concentrations were measured using the BCA protein assay kit (FUJIFILM Wako Pure Chemical) and a plate reader (Multiskan FC, ThermoFisher Scientific, USA). CPM was corrected by the amount of protein.

### Evaluation of LAT1 transport in oocytes

Supplementary Figure 1 lists the cDNAs of human transporters used in this study. First, cRNAs were synthesized *in vitro* from linearized plasmids using the mMessage mMachine Kit, polyadenylated with a Poly (A) Tailing Kit, and purified with a MEGAclear Kit (all kits: ThermoFisher Scientific, Waltham, MA, USA) according to the manufacturer’s protocols. Next, defolliculated *Xenopus* oocytes were injected with polyadenylated cRNA (25 ng/oocyte) to induce expression [24]. An equimolar amount of 4F2hc cRNA was co-injected to ensure the functional expression of LAT1 and LAT2 [24–26].

Amino acid transport was measured in the oocytes 2–4 days after cRNA injection, as previously described [24, 26–28]. Briefly, the oocytes were incubated at room temperature with 500 μL of uptake buffer (choline uptake solution: 100 mM (CH_3_)_3_N (Cl) CH_2_CH_2_OH, 2 mM KCl, 1.8 mM CaCl_2_.2H_2_O, 1 mM MgCl_2_, 5 mM HEPES, pH 7.5) containing ^211^At-PA (approximately 10kBq/oocyte). Radioactivity was determined using a gamma counter. Nine to 11 oocytes were used for each transport measurement. Two separate experiments were performed using different batches of oocytes to confirm the reproducibility of the results.

### Preparation of animals

All animal experiments were performed in compliance with the guidelines of the Institute of Experimental Animal Sciences. The protocol was approved by the Animal Care and Use Committee of the Osaka University Graduate School of Medicine. Normal ICR, normal C57BL/6, and nude mice (male) were purchased from Japan SLC Inc. (Hamamatsu, Japan). All animals were housed under a 12-h light/12-hr dark cycle, and given free access to food and water. C6 glioma cells (5.0 × 10^6^ cells) or GL-261 cells (1.0 × 10^7^ cells) were suspended in 100 μL of culture medium and Matrigel (1:1 ratio; BD Biosciences, Franklin Lakes, NJ, USA). Tumor xenograft and allograft models were prepared via the subcutaneous injection of this C6 glioma cell suspension into each immunodeficient nude mouse (*n* = 12) and the GL-261 cell suspension into each immunocompetent C57BL/6 mouse (*n* = 12), respectively [4].

### Biodistribution and evaluation of the iodine blocking effect

For the biodistribution study, para- or meta-^211^At-PA (0.53 ± 0.04 MBq) was injected into normal male ICR mice (*n* = 18, 9 weeks old, body weight = 37.7 ± 1.3 g). The mice were sacrificed by euthanasia at 1, 3, and 24 hr post-injection. For the iodine blocking study, normal male ICR mice (*n* = 6, 9 weeks old, body weight = 37.9 ± 0.9 g) were fed with 2-week iodine-restricted diet. Subsequently, the mice were divided into a blocking group (*n* = 3), which received NaI (10 mg/kg) intravenously at 24 and 3 hr before the injection of para-^211^At-PA, and a control group (*n* = 3), which did not receive NaI. The mice were euthanized 3 hr post-administration of para-^211^At-PA (0.5 MBq) and dissected.

The major organs (brain, thyroid gland, salivary gland, heart, lung, esophagus, liver, stomach, small intestine, large intestine, kidney, adrenal gland, pancreas, spleen, and testes) and the blood were collected from each mouse, and the radioactivity and weight of each sample were measured using an AccuFlex γ7000 device (Hitachi, Aloka, Japan). Radioactivity counts were normalized by calibration with a standard solution of ^211^At. The residence time (hr) was calculated using the trapezoidal method without decay correction [29].

### Evaluation of treatment effect and imaging analysis

Para-^211^At-PA was injected into mice bearing C6 glioma xenografts (*n* = 9; 15 weeks old; body weight = 23.8 ± 1.2 g) and GL261 allografts (*n* = 7; 8 weeks old; body weight = 23.6 ± 1.2 g) at 23 and 20–21 days after implantation, respectively. C6 glioma xenograft mice were divided into four groups according to the injected dose: 1 MBq (*n* = 3, 1.19 ± 0.05 MBq), 0.5 MBq (*n* = 3, 0.55 ± 0.02 MBq), 0.1 MBq (*n* = 3, 0.09 ± 0.03 MBq), and control mice without para-^211^At-PA administration (*n* = 3). GL261 allograft mice were divided into two groups: 1 MBq (*n* = 7, 1.07 ± 0.07 MBq) and control (*n* = 5). Planar imaging was performed using a gamma camera system (E-cam, Siemens) at 30 min and 3 hr after para-^211^At-PA administration. This system targeted the X-rays emitted from the daughter nuclide, ^211^Po (energy window: 79 keV ± 20%) (*6*). Image analysis was performed using AMIDE software (ver. 1.0.4). The tumor uptake was calculated as the percent injected dose (%ID). The absorbed dose (Gy) in the tumor was estimated according to a previous report with the normalization to the tumor volume in the calculation process [30]. The tumor sizes (mm^3^) were monitored using a caliper, calculated using the following elliptical sphere model equation, and compared between the injected and control groups of mice.

V = 4/3*π*a^2*b (V: volume of the tumor (mm^3^), a: shorter radius (mm) and b: longer radius (mm))

Euthanasia was applied when the following criteria were met: 1) intolerable suffering of the animal, 2) a significant decrease in activity or a marked decrease in food and water intake, and 3) the end of the observation period (up to 32 days). Euthanasia was performed via deep anesthesia induced by isoflurane inhalation.

### Statistical analysis

Comparisons of values between two groups were performed using an unpaired *t*-test. All statistical analyses were conducted using Excel 2016 (Microsoft Corp., Redmond, WA, USA). Probability values of < 0.05 were considered to indicate statistical significance. Values were reported as mean and standard deviation unless otherwise noted.

## SUPPLEMENTARY MATERIALS



## References

[1] Rajesh Y , Pal I , Banik P , Chakraborty S , Borkar SA , Dey G , Mukherjee A , Mandal M . Insights into molecular therapy of glioma: current challenges and next generation blueprint. Acta Pharmacol Sin. 2017; 38:591–613. 10.1038/aps.2016.167. 28317871 PMC5457688

[2] Stupp R , Hegi ME , Mason WP , van den Bent MJ , Taphoorn MJ , Janzer RC , Ludwin SK , Allgeier A , Fisher B , Belanger K , Hau P , Brandes AA , Gijtenbeek J , et al, and European Organisation for Research and Treatment of Cancer Brain Tumour and Radiation Oncology Groups, and National Cancer Institute of Canada Clinical Trials Group. Effects of radiotherapy with concomitant and adjuvant temozolomide versus radiotherapy alone on survival in glioblastoma in a randomised phase III study: 5-year analysis of the EORTC-NCIC trial. Lancet Oncol. 2009; 10:459–66. 10.1016/S1470-2045(09)70025-7. 19269895

[3] Kinoshita M , Arita H , Goto T , Okita Y , Isohashi K , Watabe T , Kagawa N , Fujimoto Y , Kishima H , Shimosegawa E , Hatazawa J , Hashimoto N , Yoshimine T . A novel PET index, 18F-FDG-11C-methionine uptake decoupling score, reflects glioma cell infiltration. J Nucl Med. 2012; 53:1701–08. 10.2967/jnumed.112.104992. 23000747

[4] Kratochwil C , Combs SE , Leotta K , Afshar-Oromieh A , Rieken S , Debus J , Haberkorn U , Giesel FL . Intra-individual comparison of ^18^F-FET and ^18^F-DOPA in PET imaging of recurrent brain tumors. Neuro-oncol. 2014; 16:434–40. 10.1093/neuonc/not199. 24305717 PMC3922512

[5] Watabe T , Ikeda H , Nagamori S , Wiriyasermkul P , Tanaka Y , Naka S , Kanai Y , Hagiwara K , Aoki M , Shimosegawa E , Kanai Y , Hatazawa J . ^18^F-FBPA as a tumor-specific probe of L-type amino acid transporter 1 (LAT1): a comparison study with ^18^F-FDG and 11C-Methionine PET. Eur J Nucl Med Mol Imaging. 2017; 44:321–31. 10.1007/s00259-016-3487-1. 27550420

[6] Wongthai P , Hagiwara K , Miyoshi Y , Wiriyasermkul P , Wei L , Ohgaki R , Kato I , Hamase K , Nagamori S , Kanai Y . Boronophenylalanine, a boron delivery agent for boron neutron capture therapy, is transported by ATB0,+, LAT1 and LAT2. Cancer Sci. 2015; 106:279–86. 10.1111/cas.12602. 25580517 PMC4376436

[7] Hellwig D , Ketter R , Romeike BF , Sell N , Schaefer A , Moringlane JR , Kirsch CM , Samnick S . Validation of brain tumour imaging with p-[123I]iodo-L-phenylalanine and SPECT. Eur J Nucl Med Mol Imaging. 2005; 32:1041–49. 10.1007/s00259-005-1807-y. 15902439

[8] Baum RP , Kluge A , Gildehaus FJ , Bronzel M , Schmidt K , Schuchardt C , Senftleben S , Samnick S . Systemic Endoradiotherapy with Carrier-Added 4-[(131)I]Iodo-L-Phenylalanine: Clinical Proof-of-Principle in Refractory Glioma. Nucl Med Mol Imaging. 2011; 45:299–307. 10.1007/s13139-011-0116-6. 24900021 PMC4043044

[9] Verburg FA , Sweeney R , Hänscheid H , Dießl S , Israel I , Löhr M , Vince GH , Flentje M , Reiners C , Samnick S . Patients with recurrent glioblastoma multiforme. Initial experience with p-[(131)I]iodo-L-phenylalanine and external beam radiation therapy. Nucl Med (Stuttg). 2013; 52:36–42. 10.3413/Nukmed-0510-12-06. 23303224

[10] Kruijff RM , Raavé R , Kip A , Molkenboer-Kuenen J , Morgenstern A , Bruchertseifer F , Heskamp S , Denkova AG . The *in vivo* fate of 225Ac daughter nuclides using polymersomes as a model carrier. Sci Rep. 2019; 9:11671. 10.1038/s41598-019-48298-8. 31406320 PMC6690960

[11] Watabe T , Kaneda-Nakashima K , Liu Y , Shirakami Y , Ooe K , Toyoshima A , Shimosegawa E , Fukuda M , Shinohara A , Hatazawa J . Enhancement of ^211^At Uptake via the Sodium Iodide Symporter by the Addition of Ascorbic Acid in Targeted α-Therapy of Thyroid Cancer. J Nucl Med. 2019; 60:1301–07. 10.2967/jnumed.118.222638. 30796173 PMC6735285

[12] Meyer GJ , Walte A , Sriyapureddy SR , Grote M , Krull D , Korkmaz Z , Knapp WH . Synthesis and analysis of 2-[211At]-L-phenylalanine and 4-[211At]-L-phenylalanine and their uptake in human glioma cell cultures in-vitro. Appl Radiat Isot. 2010; 68:1060–65. 10.1016/j.apradiso.2009.12.043. 20137958

[13] Borrmann N , Friedrich S , Schwabe K , Hedrich HJ , Krauss JK , Knapp WH , Nakamura M , Meyer GJ , Walte A . Systemic treatment with 4-211Atphenylalanine enhances survival of rats with intracranial glioblastoma. Nucl Med (Stuttg). 2013; 52:212–21. 10.3413/Nukmed-0580-13-05. 24036694

[14] Watanabe S , Azim MA , Nishinaka I , Sasaki I , Ohshima Y , Yamada K , Ishioka NS . A convenient and reproducible method for the synthesis of astatinated 4-[^211^At]astato-l-phenylalanine via electrophilic desilylation. Org Biomol Chem. 2018; 17:165–71. 10.1039/C8OB02394H. 30534678

[15] Aoki M , Watabe T , Nagamori S , Naka S , Ikeda H , Kongpracha P , Horitsugi G , Kanai Y , Shimosegawa E , Kanai Y , Hatazawa J . Distribution of LAT1-targeting PET tracer was independent of the tumor blood flow in rat xenograft models of C6 glioma and MIA PaCa-2. Ann Nucl Med. 2019; 33:394–403. 10.1007/s12149-019-01346-9. 30820863

[16] Bhunia S , Vangala V , Bhattacharya D , Ravuri HG , Kuncha M , Chakravarty S , Sistla R , Chaudhuri A . Large Amino Acid Transporter 1 Selective Liposomes of l-DOPA Functionalized Amphiphile for Combating Glioblastoma. Mol Pharm. 2017; 14:3834–47. 10.1021/acs.molpharmaceut.7b00569. 28958145

[17] Samnick S , Richter S , Romeike BF , Heimann A , Feiden W , Kempski O , Kirsch CM . Investigation of iodine-123-labelled amino acid derivatives for imaging cerebral gliomas: uptake in human glioma cells and evaluation in stereotactically implanted C6 glioma rats. Eur J Nucl Med. 2000; 27:1543–51. 10.1007/s002590000310. 11083545

[18] Beshr R , Isohashi K , Watabe T , Naka S , Horitsugi G , Romanov V , Kato H , Miyatake SI , Shimosegawa E , Hatazawa J . Preliminary feasibility study on differential diagnosis between radiation-induced cerebral necrosis and recurrent brain tumor by means of [^18^F]fluoro-borono-phenylalanine PET/CT. Ann Nucl Med. 2018; 32:702–08. 10.1007/s12149-018-1296-2. 30194665

[19] Horiguchi K , Tosaka M , Higuchi T , Arisaka Y , Sugawara K , Hirato J , Yokoo H , Tsushima Y , Yoshimoto Y . Clinical value of fluorine-18α-methyltyrosine PET in patients with gliomas: comparison with fluorine-18 fluorodeoxyglucose PET. EJNMMI Res. 2017; 7:50. 10.1186/s13550-017-0298-8. 28567708 PMC5451375

[20] Youland RS , Kitange GJ , Peterson TE , Pafundi DH , Ramiscal JA , Pokorny JL , Giannini C , Laack NN , Parney IF , Lowe VJ , Brinkmann DH , Sarkaria JN . The role of LAT1 in (18)F-DOPA uptake in malignant gliomas. J Neurooncol. 2013; 111:11–18. 10.1007/s11060-012-0986-1. 23086431 PMC3907171

[21] Kaira K , Oriuchi N , Imai H , Shimizu K , Yanagitani N , Sunaga N , Hisada T , Tanaka S , Ishizuka T , Kanai Y , Endou H , Nakajima T , Mori M . l-type amino acid transporter 1 and CD98 expression in primary and metastatic sites of human neoplasms. Cancer Sci. 2008; 99:2380–86. 10.1111/j.1349-7006.2008.00969.x. 19018776 PMC11159766

[22] Ikeda H , Hayashi Y , Takahashi N , Watabe T , Kanai Y , Shinohara A , Kato H , Watabe H , Shimosegawa E , Hatazawa J . Application of astatine-210: evaluation of astatine distribution and effect of pre-injected iodide in whole body of normal rats. Appl Radiat Isot. 2018; 139:251–55. 10.1016/j.apradiso.2018.05.021. 29870920

[23] Yoshimoto M , Honda N , Kurihara H , Hiroi K , Nakamura S , Ito M , Shikano N , Itami J , Fujii H . Non-invasive estimation of ^10^B-4-borono-L-phenylalanine-derived boron concentration in tumors by PET using 4-borono-2-^18^F-fluoro-phenylalanine. Cancer Sci. 2018; 109:1617–26. 10.1111/cas.13553. 29498142 PMC5980255

[24] Kanai Y , Segawa H , Miyamoto K , Uchino H , Takeda E , Endou H . Expression cloning and characterization of a transporter for large neutral amino acids activated by the heavy chain of 4F2 antigen (CD98). J Biol Chem. 1998; 273:23629–32. 10.1074/jbc.273.37.23629. 9726963

[25] Fotiadis D , Kanai Y , Palacín M . The SLC3 and SLC7 families of amino acid transporters. Mol Aspects Med. 2013; 34:139–58. 10.1016/j.mam.2012.10.007. 23506863

[26] Yanagida O , Kanai Y , Chairoungdua A , Kim DK , Segawa H , Nii T , Cha SH , Matsuo H , Fukushima J , Fukasawa Y , Tani Y , Taketani Y , Uchino H , et al. Human L-type amino acid transporter 1 (LAT1): characterization of function and expression in tumor cell lines. Biochim Biophys Acta. 2001; 1514:291–302. 10.1016/S0005-2736(01)00384-4. 11557028

[27] Wei L , Tominaga H , Ohgaki R , Wiriyasermkul P , Hagiwara K , Okuda S , Kaira K , Kato Y , Oriuchi N , Nagamori S , Kanai Y . Transport of 3-fluoro-L-α-methyl-tyrosine (FAMT) by organic ion transporters explains renal background in [(18)F]FAMT positron emission tomography. J Pharmacol Sci. 2016; 130:101–09. 10.1016/j.jphs.2016.01.001. 26887331

[28] Wei L , Tominaga H , Ohgaki R , Wiriyasermkul P , Hagiwara K , Okuda S , Kaira K , Oriuchi N , Nagamori S , Kanai Y . Specific transport of 3-fluoro-l-α-methyl-tyrosine by LAT1 explains its specificity to malignant tumors in imaging. Cancer Sci. 2016; 107:347–52. 10.1111/cas.12878. 26749017 PMC4814262

[29] Khawar A , Eppard E , Roesch F , Ahmadzadehfar H , Kürpig S , Meisenheimer M , Gaertner FC , Essler M , Bundschuh RA . Preliminary results of biodistribution and dosimetric analysis of [^68^Ga]Ga-DOTA^ZOL^: a new zoledronate-based bisphosphonate for PET/CT diagnosis of bone diseases. Ann Nucl Med. 2019; 33:404–13. 10.1007/s12149-019-01348-7. 30877560

[30] Spetz J , Rudqvist N , Forssell-Aronsson E . Biodistribution and dosimetry of free 211At, 125I- and 131I- in rats. Cancer Biother Radiopharm. 2013; 28:657–64. 10.1089/cbr.2013.1483. 23789969 PMC3793652

