# Supplementary material for: Targeted alpha therapy using astatine (211At)-labeled phenylalanine: A preclinical study in glioma bearing mice
Source: Oncotarget. 2020 Apr 14;11(15):1388–98. doi: 10.18632/oncotarget.27552 (PMC7170498; doi:10.18632/oncotarget.27552)
Supplement: Supplementary file 1 [file oncotarget-11-1388-s001.pdf]

Targeted alpha therapy using astatine (^{211}At)-labeled phenylalanine: A preclinical study in glioma bearing mice

SUPPLEMENTARY MATERIALS

Gene name	Protein name	Expression vector	Restriction enzyme site1	Enzyme for linearization	RNA polymerase	Genebank accession no.	Reference
SLC7A5	LAT1	pcDNA40	TOPO cloning2	AscI	T7	AB018009.1	Yanagida O, et al., Biochim Biophys Acta. 2001; 1514: 291-302.
SLC7A8	LAT2	pcDNA3.1(+)	NotI	XhoI	T7	AB037669.1	Pineda M, et al., J Biol Chem. 1999; 274: 19738-19744.
SLC3A2	4F2hc	pcDNA3.1(+)	HindIII, BamHI	XmaI	T7	AB018010.1	Kanai Y, et al., J Biol Chem. 1998; 273: 23629-23632.

Supplementary Figure 1: Human cDNAs and their constructs for Xenopus oocytes expression.
